# Importance of CALLY Scores in Predicting Coronary Artery Bypass Grafting Outcomes

**DOI:** 10.5761/atcs.oa.25-00156

**Published:** 2025-12-05

**Authors:** Hakkı Kursat Cetin, Tolga Demir

**Affiliations:** Department of Cardiovascular Surgery, SBU Sisli Hamidiye Etfal Training and Research Hospital, Istanbul, Turkey

**Keywords:** albumin, CALLY score, coronary artery bypass grafting, lymphocyte

## Abstract

**Purpose:**

This study aimed to clarify the importance of C-reactive protein (CRP)–albumin–lymphocyte (CALLY) index scores in predicting coronary artery bypass grafting (CABG) outcomes.

**Methods:**

Patients were divided into quartiles (Q1–Q4) based on their preoperative CALLY index values. Preoperative demographic data, laboratory parameters, operative, and postoperative outcomes were recorded.

**Results:**

The CALLY index, a composite marker incorporating CRP, albumin, and lymphocyte levels, increased progressively from Q1 to Q4, showing a statistically significant upward trend (p = 0.001). Operative and postoperative data revealed that intensive care unit (ICU) stay and hospital stay were significantly shorter in Q3 and Q4 compared to Q1 and Q2 groups (p = 0.001 for both). Furthermore, major adverse cardiac and cerebrovascular events (MACCE) rates were significantly reduced in Q3 and Q4 groups (p = 0.001), reinforcing the prognostic utility of the CALLY index. Two-year mortality also demonstrated a statistically significant reduction in the higher quartiles (p = 0.039), while in-hospital mortality did not differ significantly (p = 0.330). Operation time, cross-clamp time, and requirements for inotropic support were similar across all groups (p >0.05). The receiver-operating characteristic curve analysis demonstrated the discriminative ability of the CALLY index in predicting 2-year mortality. Area under the curve was 0.675 (95% confidence interval: 0.607–0.743), indicating moderate predictive performance.

**Conclusion:**

This study revealed that patients with higher CALLY index scores who underwent CABG had significantly shorter hospital and ICU stays. Moreover, MACCE ratio and mortality rate in the first 2 years after CABG were significantly lower in patients with higher CALLY scores.

## Introduction

Coronary artery bypass grafting (CABG) is the main surgical treatment modality for stable ischemic heart pathologies and acute coronary syndrome. Previous reports state that almost one million individuals undergo CABG procedures per year, and epidemiological studies predict an increase in the number of CABG procedures in the coming years due to the increases in diseases such as diabetes mellitus (DM) and hypertension, and the increase in the average age of society.^1)^ Although the effectiveness and reliability of CABG have been proven, the mortality rate following CABG is still up to 2%, and postoperative morbidity affects almost one in three patients who undergo CABG.^2)^ Despite the proven impact of numerous factors such as history of DM, presence of low ejection fraction, prolonged operation time, and prolonged intensive care unit (ICU) stay on CABG morbidity and mortality, noninvasive tools to predict CABG morbidity and mortality are still under investigation.^3)^

The C-reactive protein (CRP)–albumin–lymphocyte (CALLY) index is a new formula to analyze the nutritional condition, immune system status, and inflammation status of individuals. Primarily, Iida et al. developed the CALLY formula and used it for patients who were diagnosed with hepatic cancer. The authors found that the treatment success rate was significantly higher in patients with higher CALLY scores.^4)^ In another study, Güven et al. investigated the correlation between CALLY index scores and percutaneous coronary procedure outcomes and determined that patients with a CALLY index >0.7 had a significantly lower mortality rate following percutaneous coronary procedures.^5)^

A limited number of studies have evaluated the role of CALLY scores in the prediction of surgical treatment outcomes, and to our knowledge, no study has investigated the influence of CALLY scores on CABG outcomes. In the present study, we aim to clarify the importance of CALLY scores in predicting CABG outcomes.

## Materials and Methods

This retrospective observational study included patients who underwent CABG at a tertiary cardiovascular center between January 2018 and January 2024. Patients were divided into quartiles (Q1–Q4) based on their preoperative CALLY index values. Exclusion criteria included previous valve surgery, active infection, malignancy, autoimmune disorders, emergency surgery, preoperative atrial fibrillation, or missing biochemical data relevant to CALLY calculation.

Preoperative demographic data including comorbidities (hypertension, DM, chronic kidney disease), smoking status, body mass index (BMI), left ventricular ejection fraction (LVEF), and EuroSCORE II were documented. Laboratory parameters, including albumin, CRP, and lymphocyte count, were collected from routine preoperative blood tests. The CALLY index was calculated using the formula^4)^:

$$\begin{array}{rl} \text{CALLY Index }=\; & \text{Albumin }\left(\text{g}/\text{dL}\right)\times \text{Lymphocyte}\\ & \left(10^{9}/\text{L}\right)/\left(\text{CRP}\;\left[\text{mg}/\text{L}\right]\times 10\right) \end{array}$$

Additional laboratory values included triglycerides and total cholesterol. Intraoperative variables (operation and cross-clamp time, number of distal anastomoses) and postoperative outcomes (ICU stay, hospital stay, major adverse cardiac and cerebrovascular events [MACCE], hospital mortality, and 2-year mortality) were recorded.

The primary outcome was 2-year all-cause mortality. Secondary endpoints included MACCE and postoperative recovery indices. Patients were stratified into quartiles based on the CALLY index for comparative analysis of baseline characteristics, laboratory data, and clinical outcomes. This study was conducted in accordance with the principles of the Declaration of Helsinki and was approved by the institutional ethics review board.

### Statistical analysis

All statistical analyses were performed using IBM SPSS Statistics version 27.0 (IBM Corp., Armonk, NY, USA). The normality of continuous variables was assessed using the Kolmogorov–Smirnov test. Normally distributed data were expressed as mean ± standard deviation (SD) and compared using one-way analysis of variance with post hoc Bonferroni correction. Non-normally distributed variables were reported as median and interquartile range and compared using the Kruskal–Wallis test. Categorical variables were presented as frequencies and percentages, and group comparisons were performed using the chi-squared test or Fisher’s exact test, where appropriate. The predictive performance of the CALLY index and EuroSCORE II for 2-year mortality was evaluated using receiver-operating characteristic (ROC) curve analysis, and the area under the curve (AUC) was calculated with 95% confidence intervals (CIs). Multivariate logistic regression analysis was conducted to identify independent predictors of MACCE, in-hospital mortality, and 2-year mortality. Covariates included in the model were age, DM, LVEF, EuroSCORE II, and the CALLY index. Time-to-event survival data were analyzed using Kaplan–Meier survival curves, and differences between CALLY quartiles were compared using the log-rank (Mantel–Cox) test. A p value of less than 0.05 was considered statistically significant.

## Results

The baseline demographic and clinical characteristics of patients across the 4 quartile groups (Q1–Q4) showed no statistically significant differences in age, gender distribution, BMI, smoking status, LVEF, EuroSCORE II values, or distal anastomosis numbers (p >0.05 for all). However, a significant variation was observed in the prevalence of DM, which was highest in Q2 (72.2%) and significantly lower in the Q3 and Q4 groups (p = 0.001). Hypertension and chronic kidney disease prevalence did not differ significantly across groups. Preoperative demographic data of patients are summarized in **Table 1**.

**Table 1:** Comparison of demographic characteristics between groups

	Q1 (n: 155)	Q2 (n: 155)	Q3 (n: 155)	Q4 (n: 155)	p Value
Age (years)*	62.3 ± 8.5	63.2 ± 5.9	62.5 ± 6.7	62.1 ± 7.6	0.511
Gender, male (n (%))	120 (77.4%)	125 (80.6%)	115 (74.2%)	120 (77.4%)	0.605
BMI (kg/m^2^)*	27.7 ± 4.5	26.9 ± 4.6	27.3 ± 4.4	27.0 ± 4.8	0.504
Comorbidities (n (%))					
Hypertension	91 (58.7%)	95 (61.3%)	88 (56.8%)	90 (58.1%)	0.692
Diabetes mellitus	99 (63.9%)^a^	112 (72.2%)^a^	85 (54.8%)^b^	82 (52.9%)^b^	***0.001***
CKD	14 (9.0%)	15 (9.7%)	11 (7.1%)	14 (9.0%)	0.783
Smoking (n (%))	81 (52.2%)	66 (42.6%)	68 (43.9%)	69 (44.5%)	0.310
Previous PCI history (n (%))	22 (14.2%)	31 (20.0%)	25 (16.1%)	18 (11.6%)	0.218
Preoperative LVEF (%)*	49.5 ± 8.3	49.0 ± 7.8	48.3 ± 6.6	47.8 ± 7.1	0.228
EuroSCORE II**	1.55 (1.34–2.44)	1.54 (1.31–2.41)	1.41 (0.96–2.24)	1.38 (1.06–2.47)	0.095
Distal anastomosis number**	4 (2–6)	4 (1–6)	4 (2–6)	3 (1–5)	0.652

Bold-italic values denote statistically significant differences at the p <0.05 level.

Lower-case letters are used to identify the group that makes the difference. The same letters (such as a-a) indicate that there is no difference, different letters (such as a-b) indicate that there is a difference.

*Mean ± standard deviation.

**Median (IQR).

BMI: body mass index; CKD: chronic kidney disease; PCI: percutaneous coronary intervention; LVEF: left ventricular ejection fraction; IQR: interquartile range

Comparison of laboratory results between the groups is shown in **Table 2**. Albumin and lymphocyte levels were significantly higher in Q3 and Q4 compared to Q1 and Q2 groups (p = 0.001 for both). In contrast, CRP levels were significantly lower in Q3 and Q4 groups (p = 0.001). The CALLY index, a composite marker incorporating CRP, albumin, and lymphocyte levels, increased progressively from Q1 to Q4, showing a statistically significant upward trend (p = 0.001). Triglycerides and total cholesterol levels did not significantly differ among the groups (p = 0.421 and p = 0.110, respectively).

**Table 2:** Comparison of laboratory values between groups

	Q1 (n: 155)	Q2 (n: 155)	Q3 (n: 155)	Q4 (n: 155)	p Value
Blood tests					
Triglycerides (mmol/L)**	1.2 (0.9–1.8)	1.3 (1.0–1.8)	1.2 (0.9–1.9)	1.4 (1.0–2.0)	0.421
Total cholesterol (mmol/L)*	3.7 ± 1.2	3.3 ± 1.3	3.1 ± 1.2	3.8 ± 1.3	0.110
Albumin (g/dL)*	3.5 ± 1.0	3.6 ± 1.1	4.0 ± 0.8	4.0 ± 1.0	***0.001***
Lymphocytes (×10^9^/L)*	2.7 ± 1.1	3.1 ± 1.3	3.4 ± 1.1	3.7 ± 1.1	***0.001***
CRP (g/L)*	45.2 ± 20.8	14.5 ± 9.7	7.0 ± 3.0	3.3 ± 1.7	***0.001***
CALLY index**	0.23 (0.14–0.33)	0.90 (0.72–1.18)	1.91 (1.68–2.55)	4.26 (3.60–6.67)	***0.001***

Bold-italic values denote statistically significant differences at the p <0.05 level.

*Mean ± standard deviation.

**Median (IQR).

CRP: C-reactive protein; CALLY: C-reactive protein–albumin–lymphocyte

Operative and postoperative data revealed that ICU and hospital stays were significantly shorter in Q3 and Q4 groups compared to Q1 and Q2 groups (p = 0.001 for both). Furthermore, MACCE rates were significantly reduced in Q3 and Q4 groups (p = 0.001), reinforcing the prognostic utility of the CALLY index. Two-year mortality also demonstrated a statistically significant reduction in the higher quartiles (p = 0.039), while in-hospital mortality did not differ significantly (p = 0.330). Operation time, cross-clamp time, and requirements for inotropic support were similar across all groups (p >0.05) (**Table 3**).

**Table 3:** Comparison of intraoperative and postoperative data between groups

	Q1 (n: 155)	Q2 (n: 155)	Q3 (n: 155)	Q4 (n: 155)	p Value
Operation time (min)**	300 (260–340)	280 (240–320)	290 (250–330)	275 (240–320)	0.114
Cross-clamp time (min)**	81 (55–102)	72 (52–96)	72 (52–96)	67 (44–91)	0.155
Inotropic support (n (%))	30 (19.3%)	25 (16.1%)	18 (11.6%)	16 (10.3%)	0.088
ICU stay (days)*	3.4 ± 1.2^a^	3.1 ± 1.1^a^	2.4 ± 1.0^b^	2.6 ± 0.9^b^	***0.001***
Hospital stay (days)*	9.4 ± 3.1	9.1 ± 3.1	7.6 ± 2.1	7.1 ± 1.9	***0.001***
MACCE (n (%))	38 (24.5%)^a^	34 (21.9%)^a^	21 (13.5%)^b^	18 (11.6%)^b^	***0.001***
Hospital mortality (n (%))	11 (7.1%)	9 (5.8%)	8 (5.2%)	4 (2.6%)	0.330
2-year mortality (n (%))	24 (15.5%)	21 (13.6%)	11 (7.1%)	12 (7.7%)	***0.039***

Bold-italic values denote statistically significant differences at the p <0.05 level.

Lower-case letters are used to identify the group that makes the difference. The same letters (such as a-a) indicate that there is no difference, different letters (such as a-b) indicate that there is a difference.

*Mean ± standard deviation.

**Median (IQR).

ICU: intensive care unit; MACCE: major adverse cardiac and cerebrovascular events

Multivariate logistic regression analysis was performed to identify independent predictors of MACCE, in-hospital mortality, and 2-year mortality following CABG. It demonstrated that higher CALLY index scores were independently associated with lower risks of MACCE (odds ratio [OR]: 0.690, 95% CI: 0.586–0.814, p = 0.001), in-hospital mortality (OR: 0.624, 95% CI: 0.408–0.955, p = 0.030), and 2-year mortality (OR: 0.700, 95% CI: 0.564–0.869, p = 0.001). EuroSCORE II was also a significant predictor across all outcomes (p = 0.001). LVEF was inversely associated with in-hospital mortality (p = 0.038), while age was significantly associated with 2-year mortality (p = 0.001). DM was not a significant predictor in any of the models (**Table 4**).

**Table 4:** Multivariate logistic regression analysis of predictors for MACCE, in-hospital mortality, and 2-year mortality

	Odds ratio	95% CI	p Value
MACCE			
Age	0.986	0.957–1.017	0.375
Diabetes mellitus	1.409	0.910–2.183	0.125
LVEF	0.978	0.942–1.018	0.104
EuroSCORE II	1.439	1.152–1.789	***0.001***
CALLY index scores	0.690	0.586–0.814	***0.001***
Hospital mortality			
Age	0.951	0.885–1.023	0.178
Diabetes mellitus	1.387	0.515–3.735	0.517
LVEF	0.930	0.869–0.996	***0.038***
EuroSCORE II	7.307	4.219–12.656	***0.001***
CALLY index scores	0.624	0.408–0.955	***0.030***
2-year mortality			
Age	0.929	0.889–0.970	***0.001***
Diabetes mellitus	1.138	0.634–2.042	0.666
LVEF	0.987	0.951–1.026	0.517
EuroSCORE II	2.875	2.141–3.862	***0.001***
CALLY index scores	0.700	0.564–0.869	***0.001***

Bold-italic values denote statistically significant differences at the p <0.05 level.

MACCE: major adverse cardiac and cerebrovascular events; LVEF: left ventricular ejection fraction; CALLY: C-reactive protein–albumin–lymphocyte

**Fig. 1:**
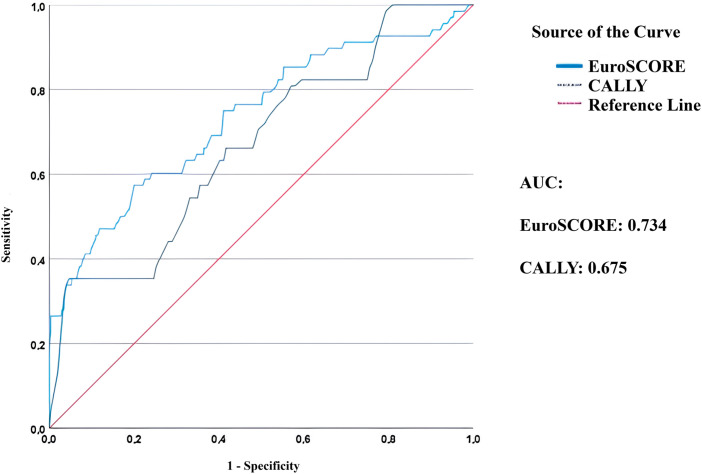
ROC curves comparing the predictive accuracy of EuroSCORE II and CALLY index for 2-year mortality. AUC: area under the curve; CALLY: C-reactive protein–albumin–lymphocyte; ROC: receiver-operating characteristic

ROC curve analysis was performed to show the predictive ability of the EuroSCORE II and the CALLY index for 2-year mortality following CABG. The AUC was 0.734 for EuroSCORE II and 0.675 for the CALLY index, indicating that both scores demonstrated acceptable discriminatory power (**Fig. 1**).

**Fig. 2:**
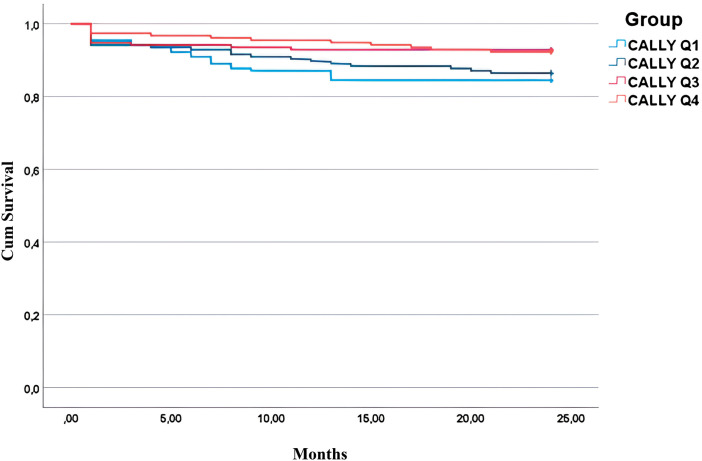
Kaplan–Meier survival curves stratified by CALLY index quartiles (Q1–Q4). CALLY: C-reactive protein–albumin–lymphocyte

Kaplan–Meier survival analysis revealed progressively longer mean survival times across increasing CALLY index quartiles. The log-rank test showed a statistically significant difference in survival distributions between the groups (χ^2^ = 8.286, degrees of freedom = 3, p = 0.040). The survival curve demonstrated that patients with higher CALLY index scores had better cumulative survival over the 2-year follow-up period, supporting the prognostic value of the CALLY index in predicting long-term outcomes after CABG (**Fig. 2**).

## Discussion

CABG is one of the most frequently performed surgical methods in the cardiovascular discipline, and the efficiency and reliability of the CABG procedure have been demonstrated by numerous studies.^6,7)^ The CALLY index has been proven effective for the prediction of disease-free survival, treatment results, and mortality in various medical conditions.^8,9)^ However, to our knowledge, the potential role of the CALLY index in predicting CABG outcomes has not yet been evaluated. Thus, we analyzed patients who underwent CABG with regard to CALLY index scores, and our findings revealed that patients with higher CALLY index scores had significantly shorter hospitalization time and ICU stay time. Moreover, the MACCE and 2-year mortality rates were significantly lower in patients with higher CALLY index scores.

Prolonged hospitalization is associated with delays in returning to daily life, increased healthcare costs, and deterioration in patient quality of life. The hospital stay length of 4000 patients was analyzed by Pontiroli et al. with regard to biochemical parameters, and the authors noted significantly longer hospitalization duration in patients with low lymphocyte count and low albumin level.^10)^ Similarly, Shibasaki et al. assessed possible factors affecting hospital stay length following cardiac surgeries and revealed that hospitalization duration significantly increased in patients with low albumin level.^11)^ Although leukocyte count and albumin levels are directly proportional to CALLY index scores, no study has investigated the effect of CALLY index scores on hospitalization following CABG. In the present research, we found significantly longer hospitalization time in patients with low CALLY index scores following CABG.

Requirement for ICU stay is common, and ICU requirements and prolonged ICU stay are associated with prolonged post-ICU hospitalization duration, blocking ICU beds for other patients, and increases in healthcare expenses. Thus, numerous studies with various results intensively analyzed possible factors that influence ICU stay length following CABG. Flegler and Paro stated that older age is the only predictive factor for prolonged ICU stay following CABG,^12)^ but in another study, Azarfarin et al. emphasized that re-exploration cases, hemodialysis, and hypotension were significantly associated with longer ICU stay after CABG.^13)^ Additionally, Kao et al. found that infection, acute renal failure, and smoking had a higher risk for longer ICU stay.^14)^ Padkins et al. demonstrated that hypoalbuminemia was significantly associated with prolonged ICU duration.^15)^ In the present study, we found an inverse proportion between CALLY index scores and ICU stay time, and patients with lower CALLY index scores had significantly longer ICU stay duration. We believe that in patients with low CALLY index scores, low albumin levels negatively affect recovery, while low lymphocyte counts negatively affect the immune response.

The main purpose of CABG is achieving cardiac revascularization and ensuring patency of cardiac vessels with minimal complications. However, CABG patients are fragile and are more likely to experience MACCE. Previous studies reported MACCE rates following CABG over a wide range. Parlar and Şaşkın analyzed predictive factors for MACCE after CABG and found that in patients with higher lymphocyte counts, MACCE rates were significantly decreased.^16)^ In another study, Schillinger et al. stated that low albumin levels predicted MACCE only in patients with a low cardiac risk profile.^17)^ Moreover, Min et al. performed a study about the correlation between CRP levels and MACCE after CABG, and the authors claimed that increased CRP resulted in significant increments in MACCE rates following CABG.^18)^ In accordance with the aforementioned results, we found significantly higher MACCE rate and 2-year mortality rate in patients with lower CALLY index scores. We believe that inadequate nutritional status and worse immune condition resulted in these situations.

The findings of this study suggest that the CALLY index, a simple composite biomarker reflecting nutritional and inflammatory status, may serve as a practical and cost-effective tool for preoperative risk stratification in patients undergoing CABG. Given its significant association with both short- and mid-term outcomes—including MACCE, hospital mortality, and 2-year mortality—the CALLY index could help clinicians identify higher-risk patients who may benefit from closer monitoring, enhanced nutritional support, or anti-inflammatory interventions. While the CALLY index showed moderate predictive performance, its AUC was lower than that of EuroSCORE II, suggesting that it may be best used as a complementary marker rather than a standalone predictor.

Although this is the first study to investigate the importance of the CALLY index for CABG results, the retrospective nature of the study is considered a limitation. In addition, we did not focus on the impact of the CALLY index on post-CABG quality of life of patients or the duration of return to daily life, which may be discussed in further studies. Also, this study did not examine the correlation between the CALLY index and health care expenses, and we believe that this may be the subject of another study. Lastly, we analyzed short- and medium-term CABG results with regard to the CALLY index; the correlation of the CALLY index with long-term CABG outcomes should be further investigated.

In conclusion, the present study revealed for the first time that patients with higher CALLY index scores who underwent CABG had significantly shorter hospitalization periods and ICU stay durations. Moreover, MACCE ratio and mortality rate in the first two years after CABG were significantly lower in patients with higher CALLY scores. Findings of the present study show that cardiovascular surgeons should pay more attention to the CALLY index scores in patients who undergo CABG, and patients with low CALLY scores who are scheduled for CABG should be informed in detail about possible undesirable conditions following surgery.
